# PySpecTrace: A Python-based Graphical User Interface for Real-Time Spectroscopy Analysis and Data Analysis

**DOI:** 10.12688/f1000research.180635.1

**Published:** 2026-07-20

**Authors:** Sigit Khoirul Anam, Joshu Leonardy, Suwardi Suwardi, Andrea Baldi, Ferry Anggoro Ardy Nugroho, Iwan Darmadi

**Affiliations:** 1 Research Center for Photonics, National Research and Innovation Agency Republic of Indonesia, Serpong, Banten, 15314, Indonesia; 2 Department of Chemistry Education, State University of Yogyakarta, Faculty of Mathematics and Natural Sciences, Yogyakarta City, Special Region of Yogyakarta, 55281, Indonesia; 3 Faculty of Electrical Engineering, Universiti Teknologi Malaysia, Skudai, Johor, 81310, Malaysia; 4 Department of Physics and Astronomy, Vrije Universiteit Amsterdam, Amsterdam, North Holland, 1081HZ, The Netherlands; 5 Department of Physics, Universitas Indonesia, Faculty of Mathematics and Natural Sciences, Depok, West Java, 16454, Indonesia; 6 Institute for Advanced Sustainable Materials Research and Technology (INA-SMART), Universitas Indonesia, Faculty of Mathematics and Natural Sciences, Depok, West Java, 16454, Indonesia

**Keywords:** sensors, plasmonics, in-situ, spectroscopy, Raman, infrared

## Abstract

**Background:**

UV–visible spectroscopy is widely used to monitor real-time transformations in material systems. However, accessible and open-source tools for real-time tracking and analysis of spectral features—including peak position, width, and intensity—remain limited. Existing workflows are often multi-step and/or reliant on proprietary software.

**Methods:**

We developed *PySpecTrace*, an open-source, Python-based graphical user interface (GUI) for real-time spectroscopy analysis. The platform integrates spectral data acquisition, preprocessing, visualization, and feature extraction within a unified and modular framework. Core functionalities include real-time tracking of peak/dip wavelength, spectral width, and intensity, as well as configurable data processing workflows. The software is designed for flexibility, enabling adaptation to generic experimental setups and time-resolved measurements.

**Results:**

PySpecTrace enables continuous monitoring and analysis of spectral evolution with minimal user intervention. Demonstration cases show its capability to track dynamic spectral changes and extract key parameters in real time, improving analysis efficiency and consistency compared to conventional post-processing approaches. In addition to real-time analysis, the platform supports post-processing of pre-recorded spectral data including feature extraction and quantitative analysis.

**Conclusions:**

PySpecTrace provides an accessible and extensible solution for real-time spectroscopy analysis, bridging experimental measurement and data processing. By offering open-source availability and reproducible workflows, it facilitates more efficient and transparent analysis of time-resolved spectroscopic data.

PySpecTrace is released under the Apache 2.0 License and is available via GitHub ^1^( https://github.com/anamsigit/pyspectrace) and Zenodo ^2^ ( https://doi.org/10.5281/zenodo.19212710), with documentation and demonstration materials provided.

## 1. Introduction

UV-visible spectroscopy is arguably the most straightforward and versatile tool for sensing and monitoring material transformation processes across various fields, including chemistry, biology, and materials science. ^3– 10^ In these applications, changes of certain features of the spectrum—for example peak shift or intensity modulation at certain wavelength—is often tracked in real-time. Observation of this spectral change in real time enables in-depth analysis, such as examining materials transformation, analyte accumulation, and reaction kinetics. ^11– 17^ For the latter, the importance of real-time UV-visible monitoring for kinetic analysis is reflected by the large number of publications in recent years. In the literature, approximately 1100 articles have been published using this approach from a short stint of January 2022 to April 2026. ^18^

To enable such a monitoring function, commercial spectrometers typically come with a proprietary software that displays spectra in real-time. However, this software is usually limited to *only* “display and record” functionality. In most cases, merely displaying the spectrum is inadequate, as often the spectral changes are too subtle and not easy to identify. Therefore, in most practice, a two-step processes are adopted: first, the spectrum is recorded using the bundled software, and second, the data is retrieved for further analysis in a separate, often homemade, program. This practice is obviously not efficient because it hinders quick decision making during the ongoing experiment.

The limitation above has stimulated the development of a real-time analysis graphical user interface (GUI) program. For instance, OceanOptics and Insplorion AB offer advanced real-time spectral analysis softwares OceanView ^®^ and Insplorer ^®^, respectively, which are equipped with visual-based programming that allows customised fitting function to track spectral changes. However, these solutions are system-tied to specific brand of spectrometers, and they limit, or entirely prohibit, the implementation of more complex fitting routines. To this date, an open-source GUI software that offers this function does not exist.

As a response, we introduce here PySpecTrace, a Python-based graphical user interface (GUI) for a real time UV-visible spectral analysis. This open and modular program supports a wide range of spectrometers, requiring only a vendor-specific application programming interface (API) module to be integrated. Most importantly, this program stands out from commercial software due to the following key features:

• A clean and easy to navigate GUI with distinct control, viewing panel and real-time analysis panels;
• Real-time fitting configuration capability *during* ongoing measurement;
• Wide range of spectrum sensitive and robust spectrum tracking toolbox for peak shift and transformation;
• Optimised peak tracking using adaptive peak fitting instead of a fixed initial guess;
• Multiple analysis displays with axis transformation functionality;
• Integrated post-processing capability;
• A spectral emulator for testing and demonstration purpose.

Specifically, PySpecTrace is designed for users who require a UV-vis real time analysis GUI that is both broadly adaptable to a wide range of spectrometers and flexible in terms of customization. In addition, the GUI is straightforward for users with minimum programming experience, while at the same time allows customization of the code for more advanced users who wish to implement more complex tracking routine tailored to specific experimental needs.

## 2. Methods

### 2.1 Implementation and architecture

PySpecTrace is structured using a Model-View-Presenter (MVP) architecture to partition the programming logic to its specific roles, i.e., Model Class, View Class and Presenter Class ^19^ ( Figure 1 and more details in Supporting Information Section I). ^36^ In detail, the Model Class retrieves and provides a spectrum from either a physical spectrometer or a built-in spectrum simulator. The View Class manages the GUI, while the Presenter class acts as a mediator between the Model Class and the View Class, coordinates data flow, and executes calculations such as referencing and fitting. Such an implementation of a well-defined architecture streamlines both maintenance and further software development processes.

**Figure 1.**
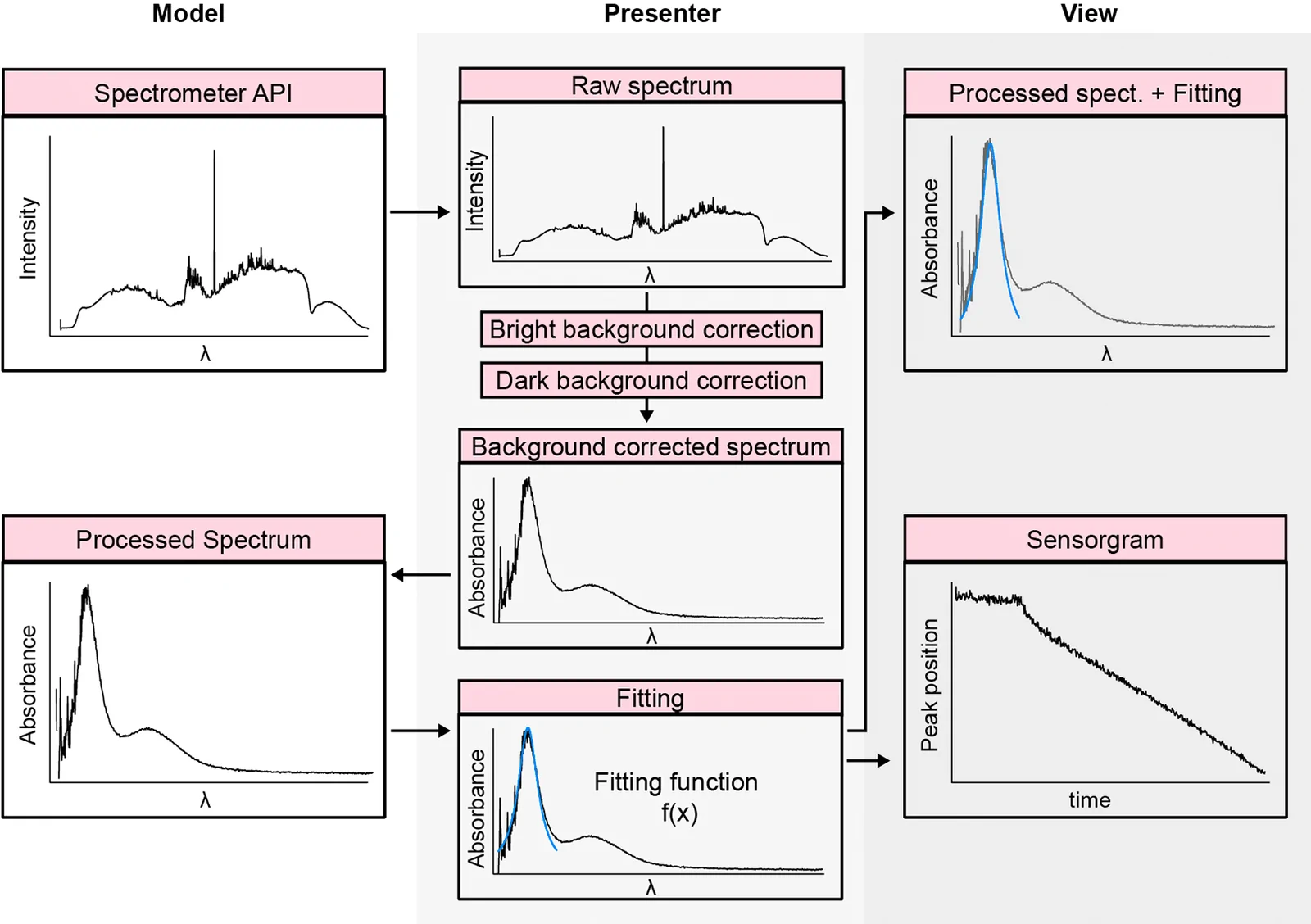
Schematic of the Model-View-Presenter (MVP) architecture behind PySpectrace. Model class retrieves raw spectra from the spectrophotometer and stores the processed spectra in preparation for fitting. Presenter class handles spectrum processing including background corrections and descriptors fitting. Finally, View class provides the platform for two types of data visualizations: (i) processed spectrum overlayed with the fittings and (ii) descriptors tracking in Sensorgram.

PySpecTrace is built upon available Python libraries as follows: (i) The PyQt5for the GUI visualisation, (ii) NumPy for the numerical computing, (iii) SciPy for the fitting routine, (iv) Pandas for tabular-based data handling, (v) Seatease for generation of simulated spectrum, and (vi) Matplotlib and PyQtgraph for the minor and the major plotting, respectively.

The data handling across MVP classes proceeds as follows: (i) The Model Class retrieves the spectral data *via* a vendor-specific API module. (ii) The Presenter processes the data (e.g., spectrum normalization with respect to bright and dark backgrounds) and subsequently stores it in the Model. This processed data can later be retrieved again for further analysis in the Presenter. (iii) Finally, all the processed data is delivered to the View Class for visualization.

To this end, PySpecTrace has been demonstrated successfully on spectrometer from Avantes ^®^, CNI Laser ^®^, and Ocean Optics ^®^. One should note, however, that an API module from the spectrometer manufacturer is required for the PySpecTrace to work. This API module is a driver that enables real-time data stream from the spectromenter. In addition to the real spectrometer data, PySpecTrace also provides a spectrometer simulator to test the PySpecTrace features without connection to a physical spectrometer. This feature enables a wide access for interested users—even ones without spectrometers—to further develop the software.

***2.1.1 Software Installation***

PySpecTrace can be installed using either (a) the Python Package Index (PyPI) or (b) manual installation from the source code repository. We recommend installation via PyPI because it automatically resolves and installs the required dependencies. Python 3.9–3.12 are recommended to ensure compatibility with all supported libraries and hardware drivers.

**(a) Installation via PyPI (recommended)**

The simplest way to install PySpecTrace is through PyPI: ^20^

pip install pyspectrace==2.0.0



(the 2.0.0 refers to the specific version 2.0.0).

After installation, the software can be launched directly from a terminal using:

pyspectrace



**(b) Manual installation from source**

Alternatively, PySpecTrace can be executed directly from the source code repository. In the directory where main.py is located, open a terminal and run:

python main.py



This command launches the PySpecTrace graphical user interface. Please note that core functionalities remain unavailable until a spectrum source is selected (see the Spectrometer Source panel in Figure 2).

**Figure 2.**
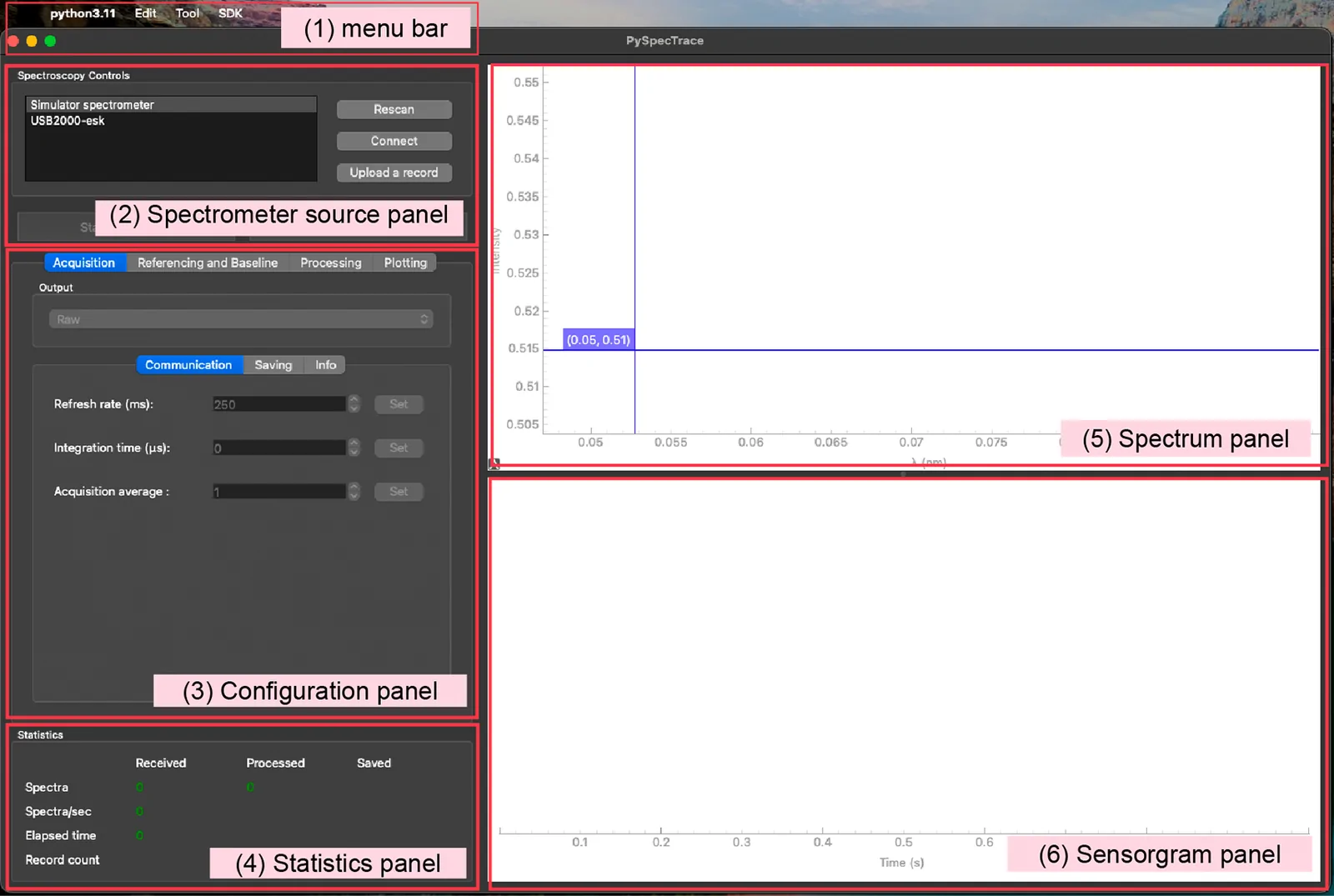
Layout of the PySpecTrace Graphical User Interface (GUI). The GUI consists of six main sections: (1) menu bar, (2) spectrometer source panel, (3) configuration panel (includes configuration for spectrometer and fitting), (4) statistics panel, (5) spectrum panel, and (6) sensorgram panel.

External spectrometers will be detected only when the SDK files (DLLs/shared libraries) are supplemented into PyspecTrace which can be uploaded via menu bar ( Figure 2) with these steps:

1. Open PySpecTrace.
2. Click SDK.
3. Choose the vendor: Ocean Optics, CNI Laser, or Avantes.
4. Select Add … DLL and choose the required .dll file.
5. Close PySpecTrace then run again

PySpecTrace stores user-provided SDK files in the user data directory. This keeps the PyPI package free from proprietary SDK files.

Detailed installation instructions, dependency management, hardware configuration, measurement procedures, and analysis workflows are beyond the scope of the present article and are therefore provided in the user manual available through Github ^1^ and Zenodo. ^36^

***2.1.2 Graphical user interface (GUI)***

To enable intuitive control yet comprehensive analysis, PySpecTrace interface is divided into five sections ( Figure 2). The Spectrometer source panel allows users to detect and connect to a spectrometer. The Configuration panel provides control for spectrometer acquisition parameters (such as integration time and spectra averaging), background referencing, fitting parameters, data recording and plotting. The Statistics panel shows real-time data stream information including rate of retrieved and processed spectra, elapsed monitoring time, and saved file counting. The Spectrum panel presents the acquired or processed spectrum in various modes such as transmission, absorbance, and reflectance. Finally, the Sensorgram displays the dynamic descriptor tracing.

***2.1.3 Software features***

PySpecTrace features several key capabilities as presented below. The corresponding detailed discussion and practical steps are documented in the provided Supporting Information and Manual, respectively (Github ^1^ and Zenodo). ^36^

i. ***Background and baseline correction***

PySpecTrace features background correction for dark and bright conditions. The dark background adjusts for the spectrometer’s electronic noise in complete darkness. The bright background, recorded when the light source is on, corrects for background materials such as the sample container and/or solvent. Both bright and dark references can be saved and reloaded, preventing the need for repeated recording. The corresponding mathematical details in determining the background spectrum are provided in the supporting documentation (Supporting Information Section II). ^36^

In addition, the program also provides baseline correction to eliminate spectrum baseline fluctuation or drift, which are common in real-time spectrum monitoring. This correction is executed by defining a baseline typically at longer wavelength where the spectrum band is flat (non-absorbing) as reference point. The baseline can be single wavelength (setting fitting order to zero) or averaged over defined wavelength range (setting fitting order to non-zero).

ii. ***Modes of spectrum output***

When both bright and dark references are recorded, the raw spectrum can be converted into different output modes: absorbance, transmission, reflection, and extinction (the formulas are described in Supporting Information Section II). The processed spectrum is displayed in a dedicated spectrum panel ( Figure 3A). If the user clears either the bright or dark spectrum, the output automatically reverts to the raw spectrum.

iii. ***Tracking of spectral evolution***

The main feature of PySpecTrace is spectral change tracking available in two options: (i) peak tracking and (ii) fixed-wavelength intensity tracking. The tracking employs a specific fitting that is displayed in real time in the Sensorgram panel ( Figure 3B).

**Figure 3.**
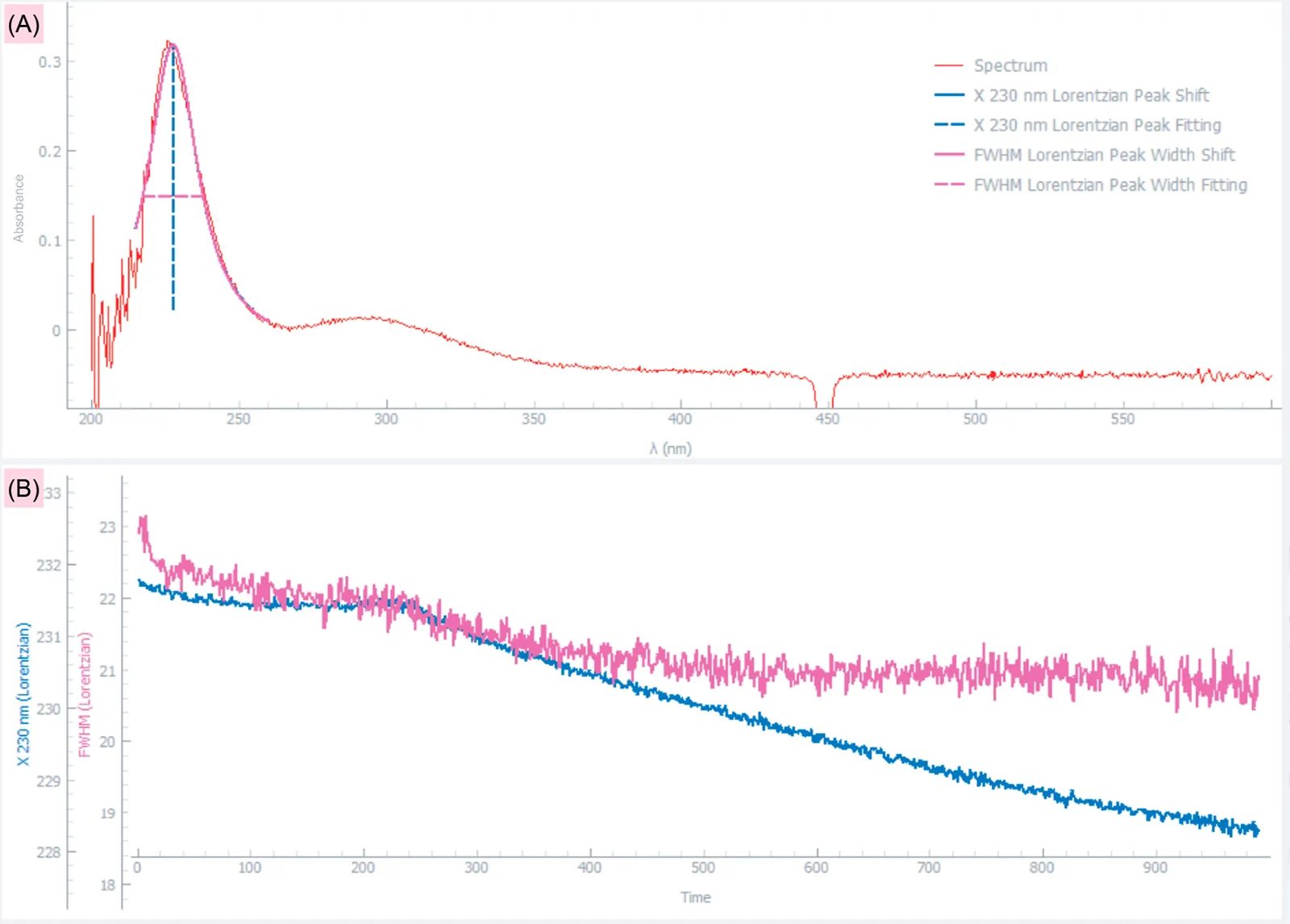
(A) Spectrum Panel showing an example of a spectrum with the corresponding peak fitting. (B) Sensorgram panel displaying the real time evolution of the peak wavelength and peak width in panel (A).

For peak position tracking, the descriptors are extracted using three kinds of optional fittings: Gaussian, Lorentzian, and polynomial. For these fittings, the user may adjust initial peak guess, fitting range, degree of polynomial and select the output descriptors. Gaussian and Lorentzian fittings return several descriptors such as peak wavelength position, peak width, and peak inflection point. The peak inflection point is determined using the definition by Chen et al. ^21^ Polynomial fitting offers an extra feature to compute the centroid (i.e. the center of mass) of the peak adopted from Dahlin et al. ^22^ Both the inflection points and centroid methods provide additional tools for tracking subtle peak transformations, which is crucial in applications reliant on peak monitoring, for example plasmonics spectroscopy ^23^ and photoluminescence. ^24^ Tracking of peak inflection points has proven more sensitive for monitoring changes in peak shoulders – a feature missed when only the peak position is tracked. Moreover, the centroid method inherently incorporate baseline correction, resulting in a signal that is more robust to baseline drift.

Another advanced feature offered by PySpecTrace is the dynamic fitting range (as opposed to the traditional static mode). In this dynamic mode, the initial peak position guess is adapted from fitting output executed one step before. This is advantageous for peak tracing where the shift is significantly larger than the peak width, which may lead to inaccurate peak fitting. This dynamic fitting mode can be activated by ticking the “Auto” box in the fitting panel (see Figure 4B). 

Last, PySpecTrace allows users to track modulation of the recorded intensity at a fixed wavelength. This tracking is executed by fitting the wavelength band around the target wavelength, followed by averaging the intensity of the fitted band. Typically, the polynomial order is set to 1 for a linear-type averaging. In addition, it is also possible to report the actual intensity of a single point wavelength without fitting by setting the fitting order to zero. All details of the available tracking methods can be found in Supporting Information Section III. ^36^

iv. ***Data visualization: Sensorgram panel***

The descriptors obtained from the tracking are displayed in the Sensorgram panel ( Figure 3B). The sensorgram can, in principle, display the real time evolution of an unlimited number of variables at once. In addition, the descriptors may independently be transformed using various standard mathematical functions (for example ln(x) , $\frac{1}{x}$ , …) typically used in chemical kinetic studies. Lastly, the y -axis scaling of each descriptor in the sensorgram is independent of the scaling of the other descriptors.

v. ***Data and graphs management***

PySpecTrace provides saving features for: (i) spectral data, (ii) sensorgram data, and (iii) generated plots. For spectral data, both raw and processed spectra are saved simultaneously. Saving raw data is encouraged to enable future unanticipated post-processing and re-analysis.

Furthermore, data saving can be paused and resumed at any time, preserving both time interval and actual timestamp. Each spectral data (including wavelength and intensity) for every data acquisition time is saved in an independent .txt file, instead of being appended to a growing collective file, to prevent potential data loss when data saving is interrupted.

For a quick visualization of the spectral evolution, PySpecTrace includes a dedicated panel that overlays spectra over time (tab ‘Spectrum multiplot’, see Manual Section IV-7 ^36^). The accumulation interval can be resumed and paused as needed. These overlaid spectra can be exported as .csv files or image formats.

Furthermore, the sensorgram tracking can also be exported as a .csv file or image formats. All recorded descriptors are organized within a single .csv file, with each descriptor allocated to separate column.

vi. ***Data post-processing
***

In addition to real-time data analysis, PySpecTrace is also equipped with a spectrum post-processing capability. This allows users to replay previously recorded spectra and apply additional tracking functions for extended analysis.

This feature also allows self-reference mode, in which each spectrum is referenced to the initial spectrum acquired at t=0 . This mode is beneficial to identify the most significant spectral changes across the wavelength range.

***2.1.4 Demonstration video***

Finally, we provided the demonstration of the software usage and the features overview in the accompanying demonstration video. ^37^ In this illustrative example, the program’s operation is presented, with its main functionalities are highlighted in Table 1.

**Table 1.** Description of the demonstration video, including timestamps and corresponding explanations of software operations and key features. The video is available in Zenodo repository. ^37^

Time (min:s)	Functionality	Description	Output/Purpose
00:00	GUI initialization	Launching the PySpecTrace graphical user interface	Start software and access main workspace
00:05	Hardware connection	Detection and connection to the spectrometer	Enable real-time data acquisition
00:09	Parameter configuration	Setting spectrometer acquisition parameters	Optimize measurement conditions
00:11	Background correction	Dark and bright background correction	Improve signal accuracy
00:24	Baseline correction	Correction of spectral baseline drift	Stabilize spectral signal
00:29	Output modes	Selection of transmission, absorbance, or reflection modes	Define spectral representation
00:34	Spectrum simulation	Simulation of spectral data	Test and validate analysis workflow
00:42	Data acquisition	Recording and saving spectral data	Store experimental measurements
00:52	Peak tracking setup	Configuration of peak tracking parameters	Prepare feature extraction
01:05	Sensorgram visualization	Real-time display of peak tracking results	Monitor spectral evolution
01:31	Adaptive peak detection	Automatic initial peak estimation (“Auto” mode)	Improve robustness of peak fitting
04:02	Multi-plot visualization	Multiple plots with independent y-axis scaling	Compare different signals simultaneously
04:35	Offline sensorgram analysis	Post-acquisition plotting and export tools	Analyze and export results
04:50	Post-processing tools	Offline spectral processing and analysis	Refine and reanalyze data
05:30	Experimental demonstration	Application in a real experimental routine	Validate practical usability

### 2.2 Operation

This program is written based on Python; therefore, it will be able to operate in Windows, Linux, and macOS. However, one should ensure that the spectrometer hardware driver also works within the operating system.

***Additional system requirements***

Input device: UV-visible-NIR spectrometer.

Computer system requirements: This program can be run on an entry level laptop or desktop PC with processor Core i3 (or equivalent) and RAM 8 GB.

***Dependencies***

Linux, Windows 10 or 11, python==3.9–3.12, numpy (2.2.5), pandas (2.2.3), matplotlib (3.10.1), pyqt5 (5.15.11), pyqtgraph (0.13.7), scipy (1.15.2), seatease (0.4.0).

## 3. Use cases

As a representative use case ( Figure 4), we demonstrate the spectral evolution of Au@Ag core–shell nanoparticle growth under laser irradiation (note that the dataset is presented solely to demonstrate the functionality of the software and does not aim to provide a comprehensive analysis of the underlying material system). The dataset used in this study is provided in the folder: /Case-study/AuAg_spectra/ within the Zenodo dataset repository ^38^ and can be directly explored by uploading the included *converted_spectrum.npz* file into the software via spectrum source panel ( Figure 2). This file was pre-generated using the built-in *Tool → Converter* function from a experimentally acquired dataset (labeled “ *Processed_spectra*” in the same folder). This ensures that users can readily reproduce the analysis workflow and explore the software functionalities using real data.

**Figure 4.**
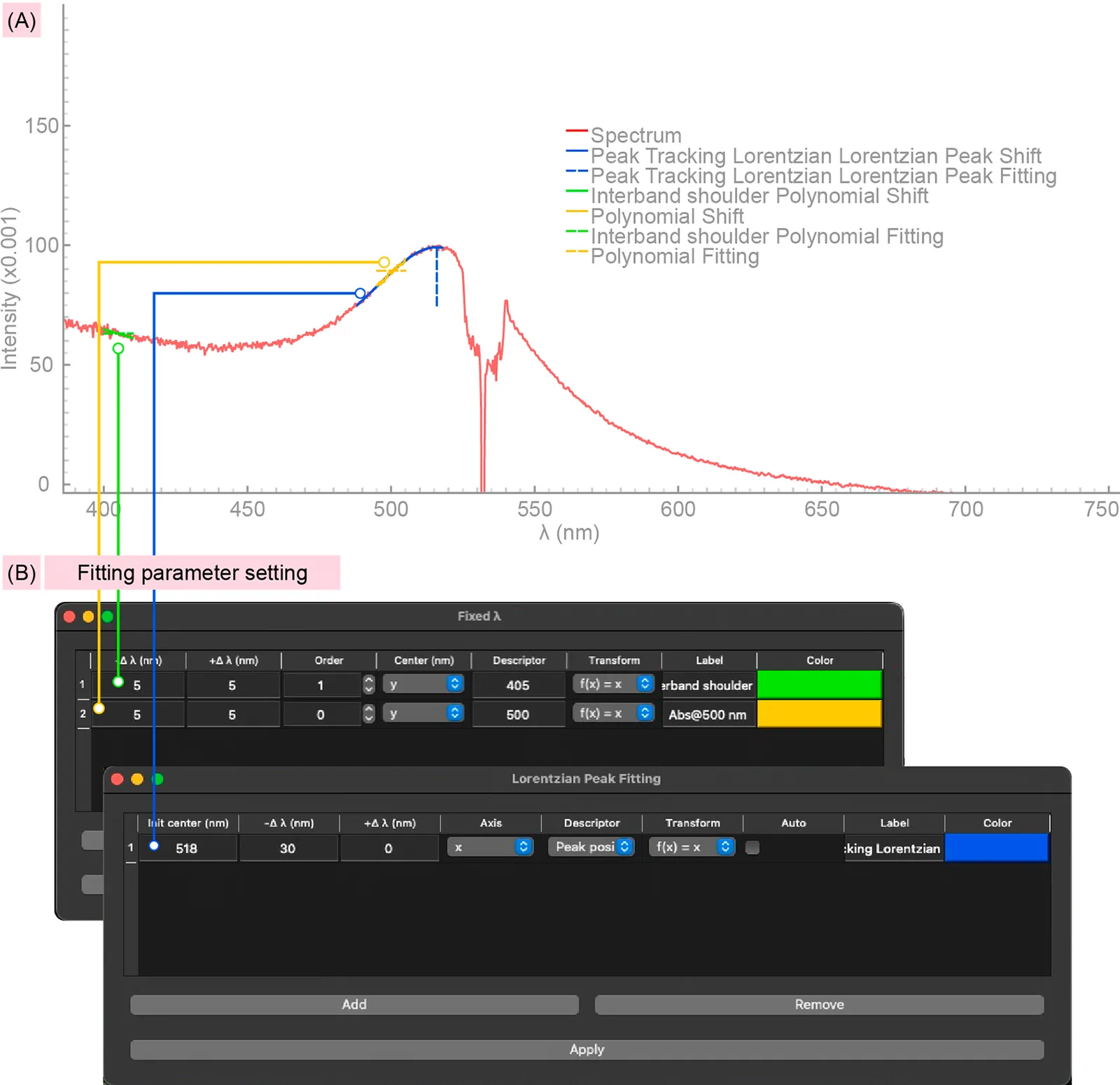
Spectral fitting case study using a notch-filtered Au@Ag spectrum. (A) Annotated screenshot of a representative spectrum showing the fitting regions, including (i) the left interband shoulder, (ii) the plasmon peak position, and (iii) the absorbance at a fixed wavelength (500 nm), used for real-time tracking. (B) Corresponding panels showing the fitting parameter settings for (i)–(iii).

This system was selected because its spectral behavior is well established. ^10, 25, 26^ During Ag shell growth, the main plasmon peak exhibits a progressive blue shift accompanied by an overall increase in absorption intensity. In addition, the short-wavelength shoulder associated with interband transitions increases simultaneously. These known trends provide a reliable physical benchmark for validating the spectral tracking performance ( Figure 4A).

This case also presents a practical analytical challenge, as the plasmon peak is partially obscured by a laser notch filter, making direct peak identification difficult. Despite this, robust peak tracking is achieved by fitting the peak slope using a Lorentzian model (initial center = 518 nm with −Δλ=30 nm), enabling reliable extraction of the peak position even within the filtered region ( Figure 4B). For the interband transition, a “non-peak” tracking approach is implemented by averaging the intensity in the 400–410 nm range using a first-order polynomial fit. Similarly, the absorbance at 500 nm is tracked using the same feature with zero-order fitting, corresponding to direct intensity extraction without additional fitting.

The evolution of the spectra shows a clear blue-shift over time ( Figure 5A). The extracted parameters—peak position, interband absorption, and absorbance at 500 nm—are summarized in the sensorgram ( Figure 5B, the movie is available in Zenodo). ^37^ All three descriptors exhibit consistent exponential trends, in agreement with previously reported observations. ^10, 25, 26^ Furthermore, these parameters show linear correlations with one another, consistent with our prior findings, supporting the internal consistency of the analysis. ^27^

**Figure 5.**
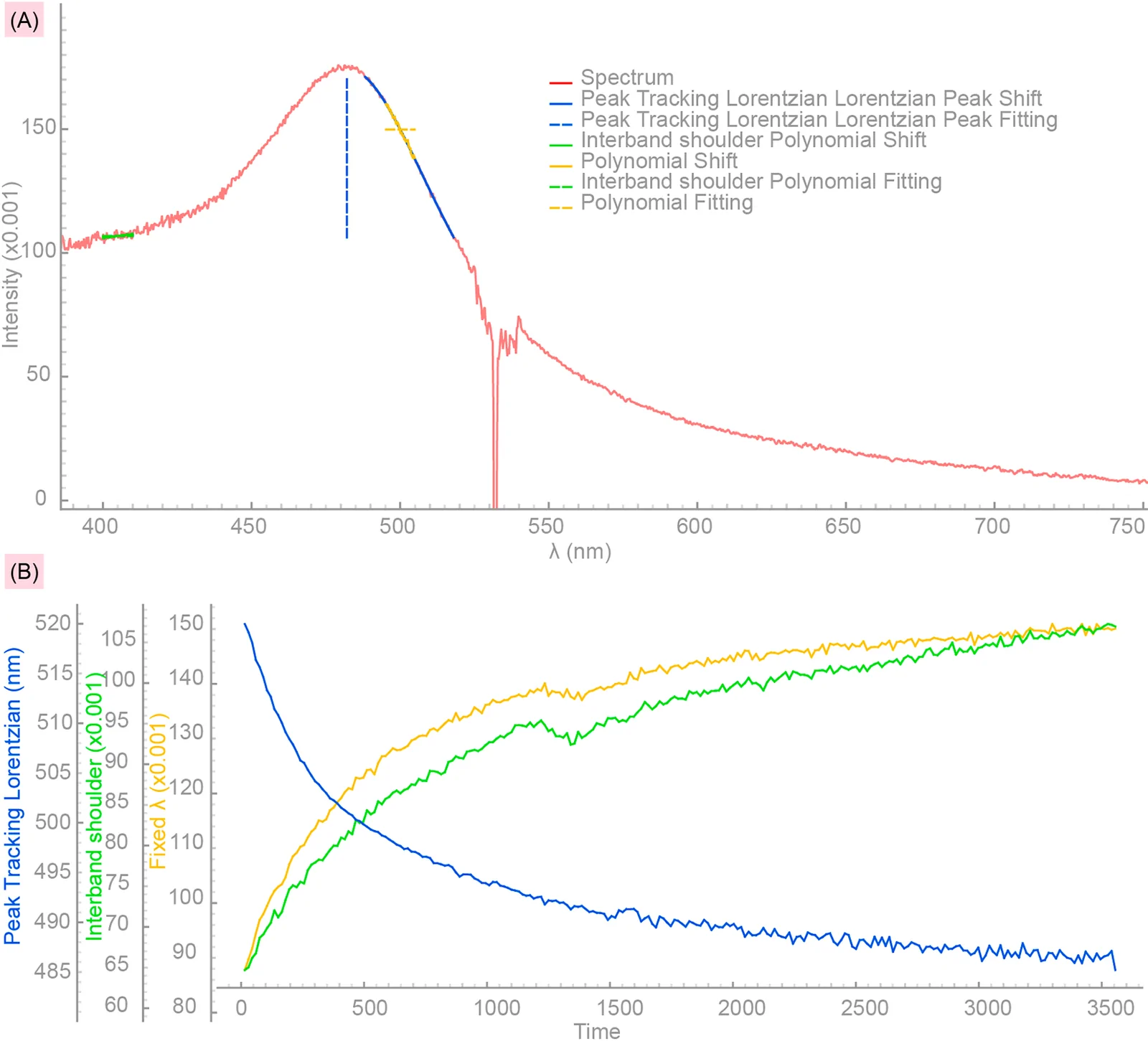
Time-resolved evolution of the Au@Ag nanoparticle spectrum. (A) Screenshot of the spectrum panel at the end of the growth process, highlighting the shift of the plasmon peak toward shorter wavelengths. (B) Screenshot of sensorgram showing the temporal evolution of (i) the interband shoulder, (ii) the peak position, and (iii) the absorbance at 500 nm.

In addition, the progression of the spectra can be visualized in overlay ( Figure 6A) using the *Plotting → Interval* function ( Figure 6B), which allows selected spectra across the time series to be displayed, facilitating inspection of spectral evolution from the initial to the final state.

**Figure 6.**
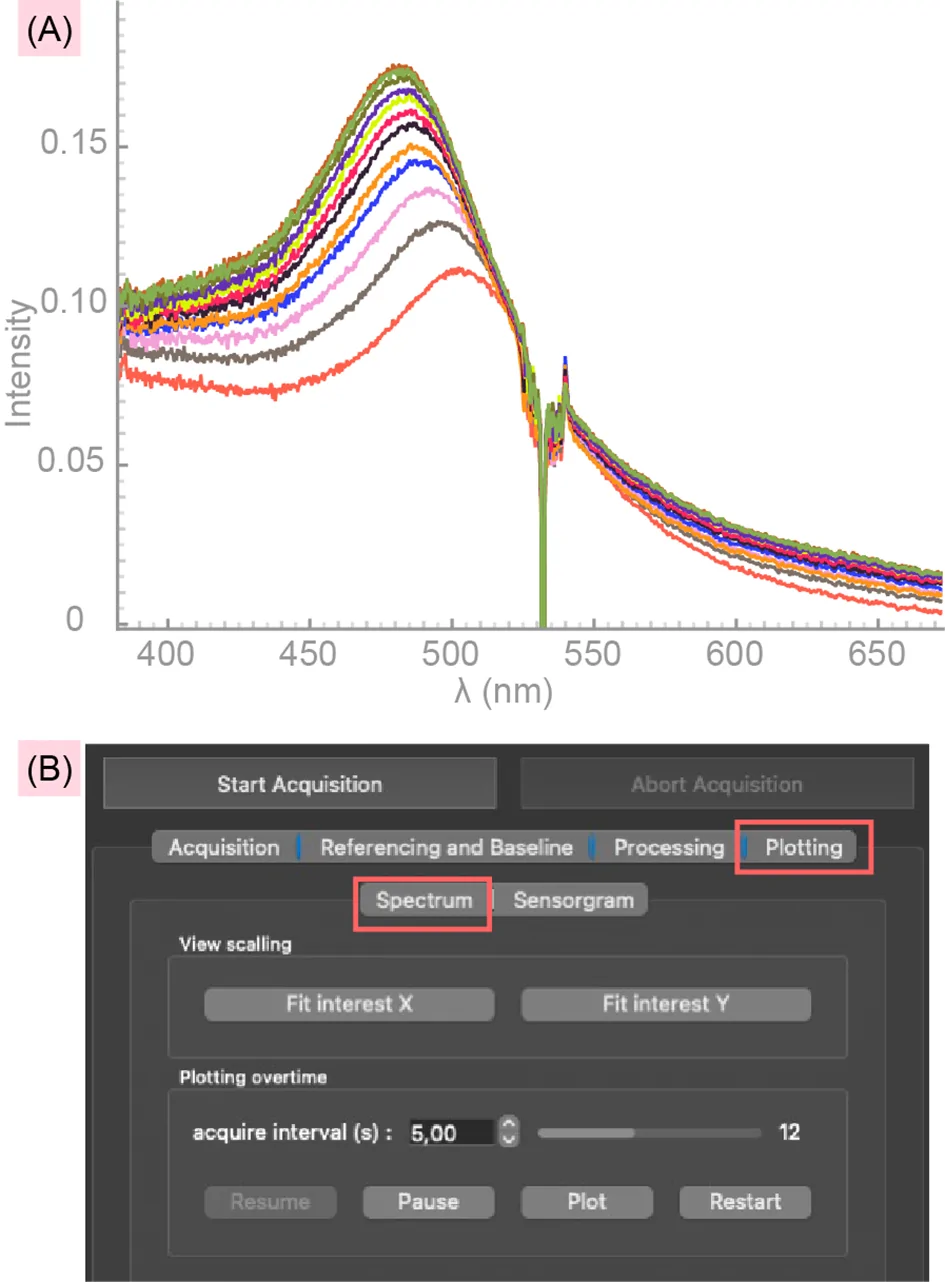
Progressive spectral evolution of Au@Ag nanoparticle growth. (A) Representative spectra spanning from the initial to the final stage. The overlayed spectra is generated using the Plotting → Spectrum tool (B) with user-defined interval selection.

Overall, this demonstration shows that the software can reliably extract and track spectral features from real experimental data, yielding results that are consistent with established physical behavior.

The code has been systematically validated on Windows platforms over the duration of May 2025–May 2026, operating continuously for several hours and across multiple days. Testing was conducted using spectrometers from three manufacturers: Ocean Optics (Maya PRO 2000, and HR4000), CNI (Aurora 4000), and Avantes (AvaSpec-ULS4096CL-EVO). Throughout these trials, the program consistently demonstrated stable performance with no critical issues were observed.

Additionally, the program can be quickly tested without the need for a physical spectrometer. The software incorporates a built-in spectrometer simulator, which enables preliminary functionality checks in the absence of hardware (see Manual for detailed instructions).

## 4. Discussion

PyspecTrace has the potential to transform current practices in kinetic study of materials transformation, where data analysis is typically performed after the entire data collection is completed. By enabling real-time, simultaneous tracking and analysis, PySpecTrace helps users make faster decisions during the experiment. This is further improved by built-in chemical kinetics models, for example zero to second-order reactions, which support immediate interpretation and accelerate discovery. As an example of the crucial real time monitoring and quick decision is the critical quenching time when performing polyol synthesis of Ag nanowires. ^28^ Another example comes from identification of Pd nanoparticle grains through analysis of its hydrogen absorption kinetics. ^17^

Beyond spectroscopy-focused research, PySpecTrace is also highly relevant for device development such as plasmonics and colorimetric sensors. ^29– 31^ Its open-access nature lowers the entry barrier for developing low-cost yet powerful optical devices. The software is designed with modularity in mind and can be readily integrated with a wide range of spectral acquisition hardware by simply adapting the supporting driver.

PySpecTrace will have a significant impact on the broad scientific community, including those working in spectroscopy, chemical sensing, and analytical instruments. In principle, the program can be adapted across the optical spectrum, extending beyond UV and visible light to techniques such as Raman and Infrared spectroscopy. ^32– 35^

Despite these advantages, several limitations should be acknowledged. The current implementation relies on predefined fitting models (e.g., Lorentzian functions), which may not fully capture more complex or overlapping spectral features. In addition, the accuracy of real-time analysis depends on signal quality and user-defined parameters, which may require careful tuning for different experimental systems. The GUI, while enhancing usability, may also limit flexibility for highly specialized or automated workflows compared to fully script-based approaches. Furthermore, the current GUI implementation depends on proprietary hardware drivers, which may restrict compatibility across different spectrometer brands. Future development will address this limitation by enabling more universal hardware integration, allowing seamless connectivity with a wider range of spectroscopic instruments.

Future developments will also focus on expanding the flexibility and analytical capabilities of the platform. This includes the integration of more advanced fitting algorithms, automated parameter optimization, and support for additional spectroscopic techniques. In particular, future developments will explore integration with experimental hardware to enable automated feedback control based on real-time spectral analysis. Such capabilities would allow dynamic adjustment of experimental conditions during operation, improving reproducibility and enabling more precise control of time-sensitive processes.

## Software availability

Source code: Github https://github.com/anamsigit/pyspectrace. ^1^

PyPi repository: https://pypi.org/project/pyspectrace. ^20^

Archived source code at time of publicatio: Zenodo https://doi.org/10.5281/zenodo.19212710. ^2^

License: Apache 2.0.

## Data Availability

1. Repository name: Dataset for Case Study of “PySpecTrace v2.0.0: Python-based Graphical User Interface (GUI) for Real-time UV-vis-NIR Spectroscopy Analysis”. https://doi.org/10.5281/zenodo.20793770 ^38^ The repository contains the following underlying data:
• Case-study.zip (this folder contains AuAg spectra obtained from real experiment data acquisition for demonstration of PySpecTrace. The dataset consists of (i) a Processed Spectra folder containing the original processed spectra in plain-text (.txt) format and (ii) a converted NumPy archive (.npz) file that can be directly loaded into PySpecTrace for reproducing the analyses presented in this article. The dataset is provided solely for demonstrating and validating the functionality of PySpecTrace and does not constitute a comprehensive study of the underlying material system. This data was used to construct Fig. 4-6). 1. Repository name: Demonstration movies of “PySpecTrace v2.0.0: Python-based Graphical User Interface (GUI) for Real-time UV-vis-NIR Spectroscopy Analysis”. https://doi.org/10.5281/zenodo.20794239 ^37^ The repository contains the following extended data:
• PySpecTrace_Case study_demo.mp4 (demonstration movie of PySpecTrace case study in AuAg spectra tracking. This data was used to construct Fig. 4-6). •
PySpecTrace_Demo_Key Features.mp4 (demonstration movie of PySpecTrace’s key features to assist spectral tracking in experimental routine. This movie is elaborated in Table 1).
2. Repository name: Manual and Supporting Information of “PySpecTrace v2.0.0: Python-based Graphical User Interface (GUI) for Real-time UV-vis-NIR Spectroscopy Analysis”. https://doi.org/10.5281/zenodo.20794452 ^36^ The repository contains the following extended data:
• PySpectrace_Manual.pdf (contains practical operational steps of PySpecTrace). • PySpectrace_Supporting Information.pdf (described detail of the software backend as well as the fitting equations). The underlying and extended data are distributed under the Creative Commons Attribution 4.0 International (CC BY 4.0). License, permitting reuse, modification, and redistribution provided appropriate credit is given to the original authors.
