# Supplementary material for: PySpecTrace: A Python-based Graphical User Interface for Real-Time Spectroscopy and Data Analysis
Source: F1000Res. 2026 Nov 12;15:1187. Originally published 2026 Jul 20. [Version 2] doi: 10.12688/f1000research.180635.2 (PMC13695712; doi:10.12688/f1000research.180635.2)
Supplement: Peer review history

# Reviewer report: Reviewer response for version 2

**DOI:** 10.5256/f1000research.213243.r541161

**Published:** 2026-11-14

**Authors:** Van Hoan Le

**Affiliations:** 1 CEA Leti, Université Grenoble Alpes, Grenoble, France

I thank the authors for their detailed responses and for the extensive revisions made in Version 2. The authors have put considerable effort into addressing the reviewers' feedback, and the manuscript is significantly strengthened as a result.

 While the major scientific and technical concerns have been satisfactorily resolved, there are a few minor and editorial points the authors should address before final indexing.

 1/ **Inconsistencies in Software Naming and Editorial Typos:** The authors intended to standardize the software name to **PySpecTrace**, but several typographical discrepancies remain in the revised text:

- **"PySpectrace"** (lowercase *t*) appears on Page 8 (Section 2.1.1 b) and Page 13 (Section 2.5), as well as in the file names listed in Extended Data 2 (Page 20).
- **"PySpectra"** (missing "-ce") has inadvertently been introduced 4 times in Section 2.3 (Page 12, line 2; Page 13, lines 3, 5, and 10).
- **"PSpecTrace"** appears in the file name in Extended Data 3 (Page 20).
- **Typo in Section 2.3 Heading (Page 12):** *"2.3 Fitting limititation"* (spelled with an extra "ti").

2/ **Installation Command (Section 2.1.1 a):** While pinning pip install pyspectrace==2.2.2 is good for reproducibility, it would be helpful to also mention the generic command “pip install pyspectrace” for general users who wish to install the latest available release (for example: When authors release a new version with updated features, users might still install the old version 2.2.2 as following instruction from this paper.

Are the conclusions about the tool and its performance adequately supported by the findings presented in the article?

Partly

Is the rationale for developing the new software tool clearly explained?

Partly

Is the description of the software tool technically sound?

Yes

Are sufficient details of the code, methods and analysis (if applicable) provided to allow replication of the software development and its use by others?

Yes

Is sufficient information provided to allow interpretation of the expected output datasets and any results generated using the tool?

Partly

Reviewer Expertise:

Characterization by vibrational spectroscopy (Raman, PL), advance materials, semiconductor, python  software developement.

I confirm that I have read this submission and believe that I have an appropriate level of expertise to confirm that it is of an acceptable scientific standard, however I have significant reservations, as outlined above.

# Reviewer report: Reviewer response for version 2

**DOI:** 10.5256/f1000research.213243.r541162

**Published:** 2026-11-13

**Authors:** Ola Kenji Forslund

**Affiliations:** 1 Department of Physics and Astronomy, Uppsala University, Uppsala, Sweden

The authors have addressed my comments and suggestions, and I believe the current version is suitable for indexing.

Are the conclusions about the tool and its performance adequately supported by the findings presented in the article?

Partly

Is the rationale for developing the new software tool clearly explained?

Yes

Is the description of the software tool technically sound?

Yes

Are sufficient details of the code, methods and analysis (if applicable) provided to allow replication of the software development and its use by others?

Yes

Is sufficient information provided to allow interpretation of the expected output datasets and any results generated using the tool?

Yes

Reviewer Expertise:

Condensed matter physics.

I confirm that I have read this submission and believe that I have an appropriate level of expertise to confirm that it is of an acceptable scientific standard.

# Reviewer report: Reviewer response for version 1

**DOI:** 10.5256/f1000research.199259.r528977

**Published:** 2026-10-26

**Authors:** Van Hoan Le

**Affiliations:** 1 CEA Leti, Université Grenoble Alpes, Grenoble, France

**Summary**: The authors present PySpecTrace, an open-source Python-based GUI designed for real-time UV-visible near-IR spectroscopy acquisition, processing and analysis. The application uses a MVP architecture and integrates spectral acquisition, preprocessing, visualization, feature extraction and post-processing within a single interface. The software provides dark- and bright-background referencing, baseline correction, conversion between spectral output modes, and several methods for tracking spectral evolution. These include Gaussian, Lorentzian and polynomial fitting; tracking of peak position, width, inflection points and centroids; fixed-wavelength intensity monitoring; and an adaptive fitting range that uses previous fitting results to update the initial peak-position estimate. The software also provides sensorgram visualization, data export, spectrum simulation and post-processing of recorded measurements. The authors demonstrate the application using spectra collected during Au@Ag core–shell nanoparticle growth. The analysis tracks the blue shift of the plasmonic peak, changes in the interband shoulder and absorbance at a fixed wavelength. The manuscript also reports testing with spectrometers from three manufacturers and provides source code, documentation and demonstration data. The software addresses a genuine experimental workflow need. Its combination of acquisition control, configurable spectral tracking and post-processing could be useful to researchers monitoring time-dependent spectral changes. Nevertheless, the manuscript requires stronger positioning against existing software and more rigorous evidence for several of its central performance claims.

**Major comments:**

 1/ The manuscript clearly explains the need for real-time spectral analysis and the limitations of conventional workflows that separate data acquisition from subsequent analysis. However, the statement in the Introduction that no open-source GUI offering this functionality exists is too broad (overstated) and insufficiently supported by a comparison with existing software.

 In addition to tools such as pyUVVIS, Spectral-GUI, python-seabreeze and SpectraFit, several other relevant open-source projects should be considered, including SpectraSorter, PyRamanGUI and SPECTROview. For example SpectraSorter published in 2022 already provides GUI-based real-time UV-Vis acquisition, spectral processing and visualization. Although it is implemented in C# and designed for specific hardware, it demonstrates that open-source real-time spectral monitoring existed before PySpecTrace. Similarly, PyRamanGUI and SPECTROview provide Python-based GUIs for spectral analysis, preprocessing, advanced baseline and multi-peak fitting features, although they do not necessarily offer the same real-time acquisition and tracking capabilities as PySpecTrace. Furthermore, the Discussion acknowledges some limitations of PySpecTrace's current fitting methods and baseline correction capabilities. The authors could therefore also consider whether existing packages provide complementary algorithms or functionalities that could be integrated into PySpecTrace in the furture versions, potentially through APIs or reusable software components, where technically feasible and compatible with their respective licenses. This could help address some of the acknowledged limitations while extending the software's capabilities.

 Thus, I recommend that the authors include a brief comparison table covering the main functionalities of these existing tools, particularly real-time acquisition, hardware compatibility, available fitting methods, baseline correction, adaptive peak tracking, extraction of spectral descriptors (e.g., inflection points and centroids), and integrated post-processing. This would help readers understand how PySpecTrace differs from existing solutions and which aspects of its functionality represent a genuine contribution.

 Rather than claiming that no comparable open-source software exists, the authors should clearly identify the specific combination of features that distinguishes PySpecTrace from existing alternatives. Such a comparison would strengthen the rationale for developing the software and provide a more accurate and convincing account of its novelty, without diminishing its potential value to the spectroscopy community.

 3/ The manuscript reports stable operation over extended periods, but provides little quantitative information about acquisition rates, processing latency or the effect of tracking multiple parameters simultaneously. Representative performance measurements would help readers assess the software's suitability for their experiments. The authors should also explain how failed or poorly constrained fits are detected and communicated to users, particularly because the adaptive fitting procedure relies on previous fitting results.

**Minor comments**

**1. **The title is somewhat redundant, as the word “analysis” appears twice. I recommend revising it slightly to make it more concise and improve its clarity.

**2. **Please qualify statements such as “unlimited” variables in the sensorgram panel. When more than approximately 10 variables are displayed simultaneously, the plots may become difficult to read and distinguish. The authors should also clarify which spectrometers and operating systems have actually been tested, distinguishing experimentally verified compatibility from expected compatibility.

**3. **The Methods section specifies ‘ **pip install pyspectrace==2.0.0**’. For general installation, ‘ **pip install pyspectrac**e’ is sufficient to install the latest compatible version available on PyPI. The authors could use this command in the main instructions while retaining the version-specific command if it is needed to reproduce the software version described in the article.

**4. **Please use the name “PySpecTrace” consistently throughout the manuscript, including figure captions. The name is currently written as “PySpec **t**race” in the caption of Figure 1 and “Py **s**pecTrace” in the installation section and Discussion.

**5. **The “built-in spectrum simulation” feature is described using different terms throughout the manuscript, including “spectral emulator” (Introduction, p. 3), “built-in spectrum simulator” and “spectrometer simulator” (Section 2.1, p. 4), and “Spectrum simulation” (Table 1, p. 9). Please use a consistent term throughout the manuscript, figures and tables. For example, “built-in spectrum simulator” would be appropriate.

**Responses to 5 questions of Peer Review Form:**

 1/ Is the rationale for developing the new software tool clearly explained? :

**Partly**: The manuscript clearly explains the need for real-time spectral analysis. However, the claim that no comparable open-source software exists is too broad and requires comparison with existing tools.

 2/ Is the description of the software tool technically sound?

**Yes **: The MVP architecture, libraries (PyQt5, NumPy, SciPy, etc.), and core functionalities (dynamic fitting, background correction, vendor-agnostic design) are well-documented and justified.  The software’s modularity and technical implementation are clearly described and aligned with best practices.

 3/ Are sufficient details provided to allow replication of the software and its use?

**Yes**

 4/ Is sufficient information provided to interpret the output datasets and results?

**Partly **: The main output parameters are explained, but information about fit reliability, uncertainty and experimental conditions remains limited.

 5/ Are the conclusions about the tool and its performance adequately supported by the findings

**Partly**: The demonstration illustrates the software's functionality, but quantitative evidence supporting its accuracy, robustness and real-time performance is insufficient.

Are the conclusions about the tool and its performance adequately supported by the findings presented in the article?

Partly

Is the rationale for developing the new software tool clearly explained?

Partly

Is the description of the software tool technically sound?

Yes

Are sufficient details of the code, methods and analysis (if applicable) provided to allow replication of the software development and its use by others?

Yes

Is sufficient information provided to allow interpretation of the expected output datasets and any results generated using the tool?

Partly

Reviewer Expertise:

Characterization by vibrational spectroscopy (Raman, PL), advance materials, semiconductor, python  software developement.

I confirm that I have read this submission and believe that I have an appropriate level of expertise to confirm that it is of an acceptable scientific standard, however I have significant reservations, as outlined above.

# Response

**Published:** 2026-10-26

**Authors:** IWAN DARMADI0906555292

Dear respected reviewer,

 We thank you for acknoledgement of our work PySpecTrace and for your constructive feedbacks.

 We agreed with your remarks and we received similar feedbacks from most reviewers here, thus these issues must be addressed seriously. In response for your concerns, we have revised the manuscript with:

 1. An acknowledgment of similar existing spectral processing GUI in the Introduction section. In this new revision, we have summarized several propietary-limited and open-source GUI with fragmented functionality in Table 1. This comparison indicates that the available tools differ in the extent to which they integrate live data acquisition with spectral-feature analysis. PySpecTrace was developed to provide these capabilities within a single open-source GUI, while recognizing that other software solutions offer overlapping functionality.

 2. We concur with the missing information about the real-time acquisition and processing performance. In the revised manuscript, we specifically dedicate Figure 5 and discussion in section 2.4 to demonstrate PySpecTrace's performance. In this demonstration, we deliberately choose high frequency refreshing rate and up to eight parallel fittings to show that PySpecTrace is capable to handle a rather heavy workload for typical UV-VIS-NIR real-time spectroscopy measurement. 

 3. For the specific information about the fitting reliability and potential fitting failure, we have now anticipated this issue by establishing a panel that in real-time displays fitting R-square. This panel is now presented in Figure 3C with description in subsection 2.1.3 part (iii)

 4. For the minor remarks about the term consistency and typos, we have addressed these in the new manuscript.

# Reviewer report: Reviewer response for version 1

**DOI:** 10.5256/f1000research.199259.r528982

**Published:** 2026-10-24

**Authors:** Anselm Hahn

**Affiliations:** 1 Max Planck Institute for Chemical Energy Conversion,, Stiftstraße, Germany

Darmadi and a coworker present PySpecTrace, a software solution for what they call real-time UV-Vis spectroscopy. The software package is fully open source, with accurate tracking via Zenodo for the software, manual, and dataset. However, inconsistencies and unresolved questions between the manuscript and the release necessitated a revision.

 Major:

- "Real-time" is not defined or referenced, and the term is used excessively.
- The software relies on outdated technologies: Windows 10, Python 3.9, and PyQt5.
- NumPy is pinned to version 1.26.4 according to the official release, whereas the manuscript lists 2.2.5. This is problematic because NumPy 2 is a major release, and no verification of numerical accuracy is provided. Running pip install pyspectrace==2.0.0 without --no-deps installs NumPy 1.x.
- It is unclear whether the described platform independence is actually provided. The manuscript repeatedly mentions DLLs; is this technically possible in a macOS or Linux environment?
- The source code uses a generic except more than 30 times, even for imports. This makes it difficult to ensure accurate execution of the software.

Minor:

- Hyphens are used excessively.
- Please link to the release tag instead of the main branch: https://github.com/anamsigit/pyspectrace/releases/tag/v2.0.0

Are the conclusions about the tool and its performance adequately supported by the findings presented in the article?

Partly

Is the rationale for developing the new software tool clearly explained?

Partly

Is the description of the software tool technically sound?

Partly

Are sufficient details of the code, methods and analysis (if applicable) provided to allow replication of the software development and its use by others?

No

Is sufficient information provided to allow interpretation of the expected output datasets and any results generated using the tool?

Partly

Reviewer Expertise:

Spectroscopy, Python, Software Development

I confirm that I have read this submission and believe that I have an appropriate level of expertise to confirm that it is of an acceptable scientific standard, however I have significant reservations, as outlined above.

# Response

**Published:** 2026-10-26

**Authors:** IWAN DARMADI0906555292

Dear respected reviewer,

 We sincerely appreciate that you devote your time to evaluate our work, not only the front-end but deeper to its back-end. We hereby respond your remarks:

 1. We acknowleged that "real-time" may lead to ambigious meaning and expectation to different community. Therefore, as a response, we have now clarified early in the introduction that real-time spectral tracking here refers to processing and displaying spectral features during continuous spectral acquisition, with each acquired spectrum analyzed before the subsequent spectrum is received under the tested acquisition conditions.

 2. Thank you for raising this important point on PySpecTrace's reliance on outdated functions. Although PySpecTrace continues to operate successfully with its current dependencies, we acknowledge that reliance on older versions may limit compatibility with users’ existing software environments. To minimize potential dependency conflicts, we now added in the manuscript a recommendion to instal PySpecTrace in an isolated Conda environment rather than modifying an existing Python environment. This recommendation has been added to subsection 2.1.1, “Software Installation.” Such isolation reduces the likelihood of conflicts with other Python packages installed on the user’s system.

 We recognize the importance of keeping software dependencies up to date. However, comprehensively modernizing the dependency stack would require substantial development and compatibility testing, and we do not consider this necessary for the current release given the demonstrated functionality of the software. We therefore regard dependency modernization as a priority for future development rather than an immediate requirement.

 3. For the DLL dependence issue, thank you for reminding us this issue. We agree that this limitation must be declared explicitly to avoid confusion. We now clarify this limitation in the section 2.5 Operation as follows:

 "This program is written in Python, enabling it to operate on Windows, Linux, and macOS platforms. However, for complete functionality, including real-time data acquisition from physical hardware, PySpectrace is currently restricted to Windows via a DLL file. Without the physical hardware connection, PySpectrace remains functional on Linux and macOS."

 4. Thank you for highlighting "generic except" aspect of the source code. We acknowledge that the use of generic exception handling can make error diagnosis and verification of execution more difficult. Although the software has operated successfully in our experimental demonstrations, we recognize that successful operation alone does not eliminate the potential risks. A systematic review and refinement of the exception-handling logic would require additional rigorous software development and testing. We therefore identify this as an area for future improvement while noting that the current implementation has supported the functionality demonstrated in the manuscript.

# Reviewer report: Reviewer response for version 1

**DOI:** 10.5256/f1000research.199259.r528978

**Published:** 2026-10-24

**Authors:** Dhanunjaya Munthala

**Affiliations:** 1 Institute of Research and Development (IRD), Suranaree University of Technology, Nakhon Ratchasima, Thailand

The manuscript presents PySpecTrace, an open-source Python GUI for real-time UV-vis-NIR spectroscopy analysis. The software integrates acquisition, preprocessing, fitting-based feature tracking, visualization, and post-processing in a modular MVP architecture. It supports multiple spectrometer vendors via vendor SDKs, offers Gaussian/Lorentzian/polynomial fitting with adaptive peak tracking, and includes a simulator for hardware-free testing. Good Architecture, useful feature set, Practical accessibility,

1. It would be helpful if the authors explained how this application is useful for analyzing basic transmittance (%) and reflectance spectra.
2.  Please include acquisition and processing rates under representative conditions (integration time, averaging, number of tracked descriptors).
3. Add a short "Limitations of validation"; consider adding a brief "Comparison with existing tools" paragraph acknowledging what the case study demonstrates.
4. Complete reference 17 and perform a full reference audit.

Are the conclusions about the tool and its performance adequately supported by the findings presented in the article?

Partly

Is the rationale for developing the new software tool clearly explained?

Yes

Is the description of the software tool technically sound?

Yes

Are sufficient details of the code, methods and analysis (if applicable) provided to allow replication of the software development and its use by others?

Yes

Is sufficient information provided to allow interpretation of the expected output datasets and any results generated using the tool?

Partly

Reviewer Expertise:

Material Science, Biosensors, Biomaterials, Bioelectronics

I confirm that I have read this submission and believe that I have an appropriate level of expertise to confirm that it is of an acceptable scientific standard.

## References

[1] : OptiEnvelope: An open-source python application for optical parameter extraction from thin-film transmission spectra. *SoftwareX* .2026;35: 10.1016/j.softx.2026.102761 10.1016/j.softx.2026.102761

# Response

**Published:** 2026-10-26

**Authors:** IWAN DARMADI0906555292

Dear respected reviewer,

 Thank you for taking your time and effort to evaluate our work and acknowledgement of the functionality leading to the approval of this manuscript.

 We appreciate your feedback and hereby we response them one-by-one ( *the reviewer's remarks are represented by quoted italics*):

*1. It would be helpful if the authors explained how this application is useful for analyzing basic transmittance (%) and reflectance spectra.*

 While for this current version PySpectra does not have explicit Transmittance nor Reflectance option, it has Transmission (%) functionality. Transmission here measure relative response light intensity in comparison to the incident light intensity. Thus, in principle, by activating this functionality, one can equally acquire Transmittance or Reflectance output.

 Nevertheless, we put your suggestions to our note for future development. 

*2. Please include acquisition and processing rates under representative conditions (integration time, averaging, number of tracked descriptors).*

 Thank you for this feedback. In response to this suggestion, we have added new demonstration on the typical averaging time, refreshing rate and number of tracked descriptors in typical data acquisition. Moreover, this test was also intented to observe PySpecTrace's processing capacity therefore stringent refreshing rate and number of tracked descriptors were chosen. As the result, PySpecTrace shows good perfomance even with this high-load data acquisition and simultaneous processing. We now present this new data in Figure 5 and include the discussion in subsection "2. 4 Data Acquisition Processing Performance."

*3. Add a short "Limitations of validation"; consider adding a brief "Comparison with existing tools" paragraph acknowledging what the case study demonstrates.*

 For limitation, we have added a new data to clarify this. Please refer to section 2.3 and Figure 5 for the fitting limitation to certain level of noise.

 In addition, for validation/comparison with existing tools, we present new demonstration of comparison between PySpecTrace fitting vs independent offline fitting in section 3.2 and Figure 9.

*4. Complete reference 17 and perform a full reference audit.*

 All references have been audited. Thank you.

# Reviewer report: Reviewer response for version 1

**DOI:** 10.5256/f1000research.199259.r528981

**Published:** 2026-10-15

**Authors:** Ola Kenji Forslund

**Affiliations:** 1 Department of Physics and Astronomy, Uppsala University, Uppsala, Sweden

The authors present PySpecTrace, an open source Python based graphical interface for real time spectroscopy analysis. The software combines spectral acquisition, processing, fitting, visualisation, and post processing within a single platform. I find the motivation clear and the software potentially useful for a broad range of experimental spectroscopy applications.

 The manuscript is generally well structured, and source code, documentation, demonstration videos, and example data is available. The Au@Ag nanoparticle growth example provides a useful illustration of the software's functionality.

 I have a few suggestions that I believe would strengthen the manuscript.

 1, Figure 5(b) provides a demonstration of how PySpecTrace tracks the evolution of the spectral parameters over time. However, I think the manuscript would benefit from a comparison with conventional offline fitting. For example, the authors could independently fit a selection of spectra from the Au@Ag dataset using standard analysis software and compare the extracted peak positions with those obtained by PySpecTrace. Plotting both results as a function of time would provide a useful validation of the automated tracking, particularly since the plasmon peak is partially obscured by the notch filter. While such a comparison would not establish the absolute accuracy of the peak positions, it would demonstrate the consistency of the automated analysis with conventional fitting methods.

 2,Since the software is intended for real time tracking, it would be helpful to clarify how unsuccessful or poorly constrained fits are handled. In particular, can users identify when the extracted peak parameters are unreliable?

 3, Since real time analysis is one of the main advantages of PySpecTrace, it would be useful to provide some quantitative information about its performance. For example, what are the typical acquisition and processing rates during an experiment, and how are these affected when multiple spectral parameters are tracked simultaneously? Even a brief description of the performance under representative experimental conditions would help readers assess the suitability of the software for their own measurements.

 4,The Introduction states that no comparable open source GUI software currently exists. As also highlighted by the first referee, this claim appears too strong given the availability of other open source spectroscopy tools. I suggest that the authors briefly compare PySpecTrace with existing alternatives and clarify which combination of features distinguishes their software. This would provide a stronger justification for its contribution without requiring the software to be entirely unprecedented.

 Overall, I consider PySpecTrace a useful contribution and believe the manuscript is suitable for indexing following clarification of these points.

Are the conclusions about the tool and its performance adequately supported by the findings presented in the article?

Partly

Is the rationale for developing the new software tool clearly explained?

Yes

Is the description of the software tool technically sound?

Yes

Are sufficient details of the code, methods and analysis (if applicable) provided to allow replication of the software development and its use by others?

Yes

Is sufficient information provided to allow interpretation of the expected output datasets and any results generated using the tool?

Yes

Reviewer Expertise:

Condensed matter physics.

I confirm that I have read this submission and believe that I have an appropriate level of expertise to confirm that it is of an acceptable scientific standard, however I have significant reservations, as outlined above.

# Response

**Published:** 2026-10-26

**Authors:** IWAN DARMADI0906555292

Dear respected reviewer,

 We sincerely thank you for the acknowledgement of  PySpecTrace value and for the constructive feedback to strengthen PySpecTrace capacity. In response to reviewer’s feedback, we hereby address the input point-by-point *(the reviewer's remarks are presented in quoted italics)*.

*"1, Figure 5(b) provides a demonstration of how PySpecTrace tracks the evolution of the spectral parameters over time. However, I think the manuscript would benefit from a comparison with conventional offline fitting. For example, the authors could independently fit a selection of spectra from the Au@Ag dataset using standard analysis software and compare the extracted peak positions with those obtained by PySpecTrace. Plotting both results as a function of time would provide a useful validation of the automated tracking, particularly since the plasmon peak is partially obscured by the notch filter. While such a comparison would not establish the absolute accuracy of the peak positions, it would demonstrate the consistency of the automated analysis with conventional fitting methods."*

 We thank the reviewer for this valuable comment. We agree that agreement with an expected qualitative trend alone is insufficient to validate the numerical fitting algorithm.

 To address this concern, we have added an independent quantitative comparison between the real-time peak-position tracking performed by PySpectra (in the case study) and an offline curve-fitting analysis independently implemented in JupyterLab (the program is available in https://doi.org/10.5281/zenodo.23555267 ).

 The additional analysis is presented in the newly added Subsection 3.2 and Figure 9 in the updated manuscript.

 The comparison demonstrates excellent agreement between the peak positions obtained using PySpectra and those determined by the independent offline reference analysis, confirming the consistency of the spectral tracking results. This additional validation provides a more rigorous assessment of the peak-position fitting performance beyond the qualitative physical trends discussed in the original manuscript.

*"2,Since the software is intended for real time tracking, it would be helpful to clarify how unsuccessful or poorly constrained fits are handled. In particular, can users identify when the extracted peak parameters are unreliable? "*

 Thank you for highlighting this crucial aspect. We concur that a dedicated information panel to inform users about the success or failure of the fitting is highly important.

 In response to this missing feature, we have now added a panel called Fitting Metrics, accessible from the Tool (in the menu bar). This panel is available since version 2.2.2 and is now accessible in the Zenodo repository.

 The Fitting Metrics panel displays the fitting metric for each descriptor and updates in real-time during the analysis. If the fitting is unsuccessful, it will be shown as NaN. For successful fitting, we display the R ^2^, which represents the confidence level of the fitting. An R ^2^ of 1 indicates a perfect fit, while a lower number indicates a poor fit.

 This new addition is now included in Figure 4C and discussed in subsection “2.1.3 Software Feature”.

*"3, Since real time analysis is one of the main advantages of PySpecTrace, it would be useful to provide some quantitative information about its performance. For example, what are the typical acquisition and processing rates during an experiment, and how are these affected when multiple spectral parameters are tracked simultaneously? Even a brief description of the performance under representative experimental conditions would help readers assess the suitability of the software for their own measurements."*

 Thank you for this important suggestion. We have added a quantitative performance assessment in Section 2.4, “Data Acquisition Processing Performance,” and Figure 5. To evaluate the workload capacity of PySpecTrace during continuous acquisition, we performed a demanding measurement using an Ocean Optics USB4000 spectrometer with a 100 ms refresh interval and 20 ms integration time. We increased the number of simultaneous spectral fittings from 2 to 5 and 8 using a desktop computer equipped with an Intel Core i7 processor (12 logical cores) and 16 GB RAM. Under all three conditions, PySpecTrace maintained an acquisition throughput of approximately 8 spectra s⁻¹, with no apparent reduction as the number of parallel fittings increased from 2 to 8. These results provide a representative quantitative assessment of acquisition performance under a deliberately high-workload condition.

*"4,The Introduction states that no comparable open source GUI software currently exists. As also highlighted by the first referee, this claim appears too strong given the availability of other open source spectroscopy tools. I suggest that the authors briefly compare PySpecTrace with existing alternatives and clarify which combination of features distinguishes their software. This would provide a stronger justification for its contribution without requiring the software to be entirely unprecedented."*

 We acknowledged that we overlooked existing similar softwares. Now we have revised the Introduction text as follows:

 The limitation above has stimulated the development of a real-time analysis graphical user interface (GUI) program. For instance, OceanOptics and Insplorion AB offer advanced real-time spectral analysis softwares OceanView ^®^ and Insplorer ^®^, respectively, which are equipped with visual-based programming that allows customised fitting function to track spectral changes. However, these solutions are system-tied to specific brand of spectrometers, and they limit, or entirely prohibit, the implementation of more complex fitting routines. On the other hand, open-source software has been developed to address the proprietary issue. To date, several open-source spectral analysis software have been independently developed. Most of the available open-source tools fall into either the real-time view-record only or spectral analysis only category. A minority of these tools offer both functionalities, but they are limited in their real-time analysis capabilities. The limitations of these software is summarized in Table 1 below.

 Table 1. Limitations of available UV-visible-NIR spectral analysis software and their comparison to PySpecTrace.

 =========

# Reviewer report: Reviewer response for version 1

**DOI:** 10.5256/f1000research.199259.r508301

**Published:** 2026-08-22

**Authors:** Abhishek Mishra

**Affiliations:** 1 McGill University, Montreal, Canada

The software is real, open, installable and solves a genuine workflow problem. The software is real, open, installable and solves a genuine workflow problem. However, the claims and conclusion made in manuscript require revision. Details are described in review comments.

**Major comments -**   

1. Many performance claims (robustness, sensitivity, efficiency, real-time capability, consistency) are asserted rather than measured, and the main novelty claim appears to be not as strong as written in the text. The Introduction states that, till date, an open-source GUI offering this function does not exist. You can find many examples such as pyUVVIS (an open-source Python UV/vis GUI with live view, recording, dark/bright reference subtraction, and Ocean Optics support), Spectral-GUI (an open-source, explicitly device-agnostic Python GUI with an abstract driver layer, threaded non-blocking acquisition and real-time plotting), python-seabreeze, SpectraFit etc. These GUIs do not provide all the features which PySpecTrace does and that can be a defensible claim. So instead of asserting the “does not exist” conclusion, a comparison can be made in terms of tool (open-source GUI?), real-time acquisition?  vendor-agnostic?  fitting models offered, inflection and centroid descriptors? adaptive window? integrated post-processing? license covering OceanView, Insplorer, and the open-source tools?
2. The AuAg case study is described as providing a reliable physical benchmark for validating the spectral tracking performance, but the benchmark is qualitative. Peak blue shifts, as expected, and three descriptors trend as expected. But agreement with an expected qualitative trend is not sufficient validation of the numerical fitting algorithm. No statement can be made about bias, precision, or the conditions under which tracking fails. Two specific claims in section 2.1.3 a) that inflection-point tracking is more sensitive to changes in peak shoulders. b) that the centroid method is more robust to baseline drift are never tested in the implementation.
3. The case study lacks experimental detail, and the deposited data lacks the raw layer.

No synthesis conditions, pre cursor concentrations, laser wavelength or power, cuvette path length, stirring, temperature, notch filter specification, integration time, spectral averaging, refresh rate or total duration are given. Even for a demonstration dataset, a reader needs enough to judge whether the spectral behavior is what the authors say it is, particularly since the physical trends are being used as the benchmark for the tracking.

1. The case study rests on a one-sided fit, and this is not acknowledged. Figure4B shows the Lorentzian window as −Δλ = 30 nm, +Δλ = 0 nm about a 518 nm centre i.e. only the blue flank is fitted, because the red side is destroyed by the notch filter. The text describes this simply as "Δλ = 30 nm", which reads as a symmetric window.
2. The Discussion refers to "built-in chemical kinetics models, for example zero to second-order reactions". There is no mention of this in the Methods, the Introduction feature list, any figure, Table 1, or the Use cases.

**Minor Comments**

1. Title. "Real-Time Spectroscopy Analysis and Data Analysis" is internally redundant, "analysis" appears twice
2. The software's own name is spelled three different ways: PySpecTrace (most places), PySpectrace (Figure 1 caption), and PyspecTrace (2.1.1, "supplemented into PyspecTrace"; Discussion, opening sentence "PyspecTrace has the potential…"). Standardize to PySpecTrace throughout, including figure captions.
3. The simulator is called a "spectral emulator" in the Introduction bullet list, a "built-in spectrum simulator" in 2.1, a "spectrometer simulator" in 2.1 and 3, and "Spectrum simulation" in Table 1. Pick one term and use it everywhere
4. "from a short stint of January 2022 to April 2026”, “short stint" is the wrong register for a literature-count statement, and a four-year-plus window is not short. Also, the sentence before refers to "recent years", so the two do not sit well together.
5. Ref 17 (Alekseeva et al., "Grain-Growth Mediated Hydrogen Sorption Kinetics and Compensation Effect in Single Pd Nanoparticles", 2021) has no journal, volume or pages. Please complete it. Similar minor mistakes in references throughout, please check before final submission.

Are the conclusions about the tool and its performance adequately supported by the findings presented in the article?

Partly

Is the rationale for developing the new software tool clearly explained?

Yes

Is the description of the software tool technically sound?

Yes

Are sufficient details of the code, methods and analysis (if applicable) provided to allow replication of the software development and its use by others?

Yes

Is sufficient information provided to allow interpretation of the expected output datasets and any results generated using the tool?

Yes

Reviewer Expertise:

Bioprocess Monitoring, Bioengineering, Spectroscopy

I confirm that I have read this submission and believe that I have an appropriate level of expertise to confirm that it is of an acceptable scientific standard, however I have significant reservations, as outlined above.

# Response

**Published:** 2026-10-25

**Authors:** IWAN DARMADI0906555292

Dear Respected Reviewer,

 Thank you for acknowledging the utility of PySpecTrace. We sincerely appreciate your time and effort in providing us with constructive feedback. Below, we address your suggestion point-by-point ( *reviewer's note is represented with italics*).

*The software is real, open, installable and solves a genuine workflow problem. The software is real, open, installable and solves a genuine workflow problem. However, the claims and conclusion made in manuscript require revision. Details are described in review comments.*

*Many performance claims (robustness, sensitivity, efficiency, real-time capability, consistency) are asserted rather than measured.*

 We agree with the reviewer’s concern and have revised the manuscript to clarify and, where possible, quantitatively evaluate the previously stated performance characteristics.

- **Sensitivity.** We evaluated the fitting sensitivity of PySpecTrace using synthetic Gaussian peaks under different noise levels. The analysis showed that peak tracking remained successful at a signal-to-noise ratio (SNR) of approximately 33%. This quantitative assessment has been added to Figure 4 and discussed in Section 2.3. We have also revised the text to describe this result as an experimentally evaluated operating limit rather than making a general claim about sensitivity.
- **Robustness.** We added a fitting-parameter sensitivity analysis to examine whether the fitting procedure remains stable when the fitting parameters are varied within the relatively relaxed ranges used by PySpecTrace. The results are presented in Figure 10 and discussed in Section 3.3. This analysis provides quantitative evidence of the dependence of the fitting outcome on the selected parameters and supports the robustness of the fitting procedure under the tested conditions.
- **Efficiency.** We have clarified that our use of “efficiency” refers primarily to workflow efficiency rather than computational efficiency. PySpecTrace integrates spectral acquisition, real-time feature tracking, and data analysis within a single software environment, thereby eliminating the need to use separate software tools for simultaneous spectral recording and dynamic spectral-feature analysis. We have revised the manuscript to make this meaning explicit.
- **Real-time capability.** To avoid ambiguity, we now define real-time operation in the Introduction as the capability to process and analyze an acquired spectrum before the subsequent acquisition. In addition, the statistical panel provides live reporting of the incoming data stream and processing. We further quantified this capability under demanding acquisition conditions by measuring spectral-processing throughput with multiple simultaneous fitting tasks. As shown in Figure 5 (Section 2.4), PySpecTrace maintained an acquisition throughput of approximately 8 spectra s⁻¹ while processing up to eight simultaneous fitting tasks.
- **Consistency.** We independently reproduced the fitting analysis for the Au@Ag growth case study using a Python-based offline curve_fit implementation and compared the resulting peak-tracking values with those obtained from PySpecTrace. The results, presented in Figure 9 and discussed in Section 3.2, show close agreement between the two approaches, providing an independent assessment of the consistency of PySpecTrace fitting results.

*The main novelty claim appears to be not as strong as written in the text. The Introduction states that, till date, an open-source GUI offering this function does not exist. You can find many examples such as pyUVVIS (an open-source Python UV/vis GUI with live view, recording, dark/bright reference subtraction, and Ocean Optics support), Spectral-GUI (an open-source, explicitly device-agnostic Python GUI with an abstract driver layer, threaded non-blocking acquisition and real-time plotting), python-seabreeze, SpectraFit etc. These GUIs do not provide all the features which PySpecTrace does and that can be a defensible claim. So instead of asserting the “does not exist” conclusion, a comparison can be made in terms of tool (open-source GUI?), real-time acquisition?  vendor-agnostic?  fitting models offered, inflection and centroid descriptors? adaptive window? integrated post-processing? license covering OceanView, Insplorer, and the open-source tools?*

 We acknowledged our unawarness of existing similar softwares. Now we have revised the introduction text as follows:

 The limitation above has stimulated the development of a real-time analysis graphical user interface (GUI) program. For instance, OceanOptics and Insplorion AB offer advanced real-time spectral analysis softwares OceanView ^®^ and Insplorer ^®^, respectively, which are equipped with visual-based programming that allows customised fitting function to track spectral changes. However, these solutions are system-tied to specific brand of spectrometers, and they limit, or entirely prohibit, the implementation of more complex fitting routines. On the other hand, open-source software has been developed to address the proprietary issue. To date, several open-source spectral analysis software have been independently developed. Most of the available open-source tools fall into either the real-time view-record only or spectral analysis only category. A minority of these tools offer both functionalities, but they are limited in their real-time analysis capabilities. The limitations of these software is summarized in Table 1 below. (Table 1. Limitations of available UV-visible-NIR spectral analysis software and their comparison to PySpecTrace.)

*The AuAg case study is described as providing a reliable physical benchmark for validating the spectral tracking performance, but the benchmark is qualitative. Peak blue shifts, as expected, and three descriptors trend as expected. But agreement with an expected qualitative trend is not sufficient validation of the numerical fitting algorithm.*

 We thank the reviewer for this valuable comment. We agree that agreement with an expected qualitative trend alone is insufficient to validate the numerical fitting algorithm.

 To address this concern, we have added an independent quantitative comparison between the real-time peak-position tracking performed by PySpectra, as presented in Figures 9  and an offline curve-fitting analysis independently implemented in JupyterLab (the program is available in https://doi.org/10.5281/zenodo.23555267 ). The additional analysis is presented in the newly added Subsection 3.2 and Figure 9.

 The comparison demonstrates excellent agreement between the peak positions obtained using PySpectra and those determined by the independent offline reference analysis, confirming the consistency of the spectral tracking results. This additional validation provides a more rigorous assessment of the peak-position fitting performance beyond the qualitative physical trends discussed in the original manuscript.

*No statement can be made about bias, precision, or the conditions under which tracking fails.*

 We thank the reviewer for pointing out that the original manuscript did not provide sufficient quantitative discussion of potential bias, precision, or the conditions under which peak tracking may fail. We agree that these aspects are important for assessing the reliability and practical limitations of the tracking algorithm.

 Regarding **bias and precision**, the fitted peak position can be affected by the selection of fitting parameters, particularly the fitting window. To examine the sensitivity of the peak-position tracking to this parameter, we have therefore added an additional analysis in **Section 3.3 and Figure 10**, in which the fitting range is systematically varied around the optimized value. In this analysis, the optimized fitting window of 30 nm was varied from 20 to 60 nm, allowing us to assess the effect of both narrower and wider fitting windows on the resulting peak positions.

 The results show that, over a relatively broad range of fitting windows (25–50 nm), the tracked peak positions deviate only slightly from the reference peak positions. This indicates that the peak-position determination is relatively tolerant to moderate deviations from the optimized fitting window. Importantly, the fitting window investigated here is relatively broad in the context of spectral peak analysis, suggesting that small inaccuracies in the initial selection of the fitting range do not substantially affect the resulting peak-position tracking.

 Furthermore, even when the fitting window deviates substantially from the optimized value, the overall temporal trend of the peak movement remains recognizable with only minor errors. This minor errors  can be revised later using the built-in post-processing function that allows the recorded data to be reanalyzed after acquisition. Thus, an initially non-optimal fitting parameter does not necessarily compromise the recorded measurement, provided that the raw spectral data have been recorded. The fitting parameters can subsequently be adjusted during post-processing to obtain improved peak tracking.

 Regarding the **conditions under which tracking may fail**, we have clarified several relevant scenarios. First, tracking performance can deteriorate when the signal-to-noise ratio becomes too low, as demonstrated by the synthetic noise analysis discussed above. Second, the automatic tracking mode uses the peak position obtained from the preceding spectrum as the initial estimate for the subsequent spectrum. Consequently, an unexpected spectral anomaly (where the peak suddently disappear) may cause fail fitting process, interrupting the subsequent tracking sequence. This limitation and the corresponding behavior have now been explicitly described in **Section 2.1.1 (iii)**. Importantly, such a tracking failure does not interrupt the underlying spectral acquisition; the raw spectra continue to be recorded and can subsequently be reanalyzed with the post-processing functionality (note that the Auto feature should be deactivated for this recalculation).

 Third, tracking reliability is inherently related to the quality of the curve fitting itself. To make this information directly accessible to users, we have added the **coefficient of determination (R²)** of the fitted peak to the spectrum panel in the updated version of PySpectra. The R² value is now readily visible during analysis, providing an immediate indication of the quality of the current peak fit and allowing users to identify spectra for which the fitted peak position may be unreliable.

 Together, these additional analyses and software improvements provide a more explicit characterization of the robustness, limitations, and failure conditions of PySpectra’s peak-tracking algorithm.

*Two specific claims in section 2.1.3 a) that inflection-point tracking is more sensitive to changes in peak shoulders. b) that the centroid method is more robust to baseline drift are never tested in the implementation.*

 We apologize for the ambiguity in the original manuscript. The statements regarding the higher sensitivity of inflection-point tracking to changes in peak shoulders and the greater robustness of the centroid method to baseline drift were intended to summarize findings reported in the relevant literature, rather than claims derived from our own implementation or experimental validation.

 To avoid implying that these characteristics were independently demonstrated in the present study, we have revised Section 2.1.3 to explicitly attribute these observations to the systematic studies reported in the cited references.

*The case study lacks experimental detail, and the deposited data lacks the raw layer. No synthesis conditions, pre cursor concentrations, laser wavelength or power, cuvette path length, stirring, temperature, notch filter specification, integration time, spectral averaging, refresh rate or total duration are given. Even for a demonstration dataset, a reader needs enough to judge whether the spectral behavior is what the authors say it is, particularly since the physical trends are being used as the benchmark for the tracking.*

 We understand the experimental detail may be relevant to assess the peak tracking result. However, we believe that the experimental detail is not necessary to present in the main text as it will deviate the discussion focusing on the software itself. Moreover, the experimental detail is adopted from from published articles elsewhere:

- Nguyen Q, Baldi A: Size Matters: d-Band Holes Drive Plasmonic Chemistry in Gold. *Nano Lett.*2025;25:14704–14709. 40980841 10.1021/acs.nanolett.5c03849 PMC12512193
- Kamarudheen R, Castellanos GW, Kamp LPJ, et al.: Quantifying Photothermal and Hot Charge Carrier Effects in Plasmon-Driven Nanoparticle Syntheses. *ACS Nano.* 2018;12:8447–8455. 30071160 10.1021/acsnano.8b03929
- Kamarudheen R, Aalbers GJW, Hamans RF, et al.: Distinguishing among All Possible Activation Mechanisms of a Plasmon-Driven Chemical Reaction. *ACS Energy Lett.* 2020;5:2605–2613. 10.1021/acsenergylett.0c00989

We have now explicitly mention these references in the main text.

*The case study rests on a one-sided fit, and this is not acknowledged. Figure4B shows the Lorentzian window as −Δλ = 30 nm, +Δλ = 0 nm about a 518 nm centre i.e. only the blue flank is fitted, because the red side is destroyed by the notch filter. The text describes this simply as "Δλ = 30 nm", which reads as a symmetric window.*

 We have clarified this point in the revised manuscript and explicitly described the fitting window as a one-sided fitting range. Furthermore, we have added an analysis in Figures 9 and 10 to examine the effect of the fitting-window selection on the retrieved peak position. The results show that the one-sided fitting used in this case does not produce a substantial deviation in the fitted peak position compared with the reference analysis. These additional results provide quantitative support that the one-sided fitting necessitated by the notch-filter configuration does not substantially compromise the peak-position tracking.

*The Discussion refers to "built-in chemical kinetics models, for example zero to second-order reactions". There is no mention of this in the Methods, the Introduction feature list, any figure, Table 1, or the Use cases.*

 Thank you for pointing out this unproven claim. We have now remove this statement from the text.

***Minor Comments***

1. *Title. "Real-Time Spectroscopy Analysis and Data Analysis" is internally redundant, "analysis" appears twice*

We acknowlegde this overlook and completely agree with the author to revise the title.

1. *The software's own name is spelled three different ways: PySpecTrace (most places), PySpectrace (Figure 1 caption), and PyspecTrace (2.1.1, "supplemented into PyspecTrace"; Discussion, opening sentence "PyspecTrace has the potential…"). Standardize to PySpecTrace throughout, including figure captions.*

We thank the reviewer to spot on this inconsistency. We have now implement the correction by standardizing the term to be “PySpecTrace”.

1. *The simulator is called a "spectral emulator" in the Introduction bullet list, a "built-in spectrum simulator" in 2.1, a "spectrometer simulator" in 2.1 and 3, and "Spectrum simulation" in Table 1. Pick one term and use it everywhere*

We thank the reviewer to spot on this inconsistency in detail. We have now implemented the correction by standardizing the term to be “Spectral simulator” throughout the text.

1. *Ref 17 (Alekseeva et al., "Grain-Growth Mediated Hydrogen Sorption Kinetics and Compensation Effect in Single Pd Nanoparticles", 2021) has no journal, volume or pages. Please complete it. Similar minor mistakes in references throughout, please check before final submission.*

We have now corrected the references format.
